# Using Expression and Genotype to Predict Drug Response in Yeast

**DOI:** 10.1371/journal.pone.0006907

**Published:** 2009-09-04

**Authors:** Douglas M. Ruderfer, David C. Roberts, Stuart L. Schreiber, Ethan O. Perlstein, Leonid Kruglyak

**Affiliations:** 1 Center for Human Genetic Research, Massachusetts General Hospital, Boston, Massachusetts, United States of America; 2 Eli & Edythe L. Broad Institute of Harvard and MIT, Cambridge, Massachusetts, United States of America; 3 Theoretical Division and Center for Nonlinear Studies, Los Alamos National Laboratory, Los Alamos, New Mexico, United States of America; 4 Department of Chemistry and Chemical Biology, Howard Hughes Medical Institute, Cambridge, Massachusetts, United States of America; 5 Lewis-Sigler Institute for Integrative Genomics, Princeton University, Princeton, New Jersey, United States of America; 6 Department of Ecology and Evolutionary Biology, Princeton University, Princeton, New Jersey, United States of America; Victor Chang Cardiac Research Institute (VCCRI), Australia

## Abstract

Personalized, or genomic, medicine entails tailoring pharmacological therapies according to individual genetic variation at genomic loci encoding proteins in drug-response pathways. It has been previously shown that steady-state mRNA expression can be used to predict the drug response (i.e., sensitivity or resistance) of non-genotyped mammalian cancer cell lines to chemotherapeutic agents. In a real-world setting, clinicians would have access to both steady-state expression levels of patient tissue(s) and a patient's genotypic profile, and yet the predictive power of transcripts versus markers is not well understood. We have previously shown that a collection of genotyped and expression-profiled yeast strains can provide a model for personalized medicine. Here we compare the predictive power of 6,229 steady-state mRNA transcript levels and 2,894 genotyped markers using a pattern recognition algorithm. We were able to predict with over 70% accuracy the drug sensitivity of 104 individual genotyped yeast strains derived from a cross between a laboratory strain and a wild isolate. We observe that, independently of drug mechanism of action, both transcripts and markers can accurately predict drug response. Marker-based prediction is usually more accurate than transcript-based prediction, likely reflecting the genetic determination of gene expression in this cross.

## Introduction

Realizing the promise of personalized medicine – a rational approach to tailoring pharmacological therapy to individual patients – is an area of intense research [1], [2]. One of the central experimental challenges of personalized medicine is to identify physiological correlates (i.e., biomarkers) of individual genetic variation that would serve as reliable diagnostic indicators of (desired) drug response and (undesired) side effects [3]–[5]. The most obvious diagnostic indicator of drug response is genetic variation itself in the form of single-nucleotide polymorphisms (SNPs), insertions or deletions, or gross chromosomal rearrangements, which can be catalogued by genotyping techniques. Genetic variation in genes known to modulate drug response in general, such as the ABC family of xenobiotic transporters, or the cytochrome P450 detoxification enzymes, has been successfully correlated to clinical outcomes of drug therapy [6]–[11]. Additionally, in candidate-gene approaches, polymorphisms in the molecular targets of drugs (or downstream pathway components) have also been correlated with clinical outcome in cancer and in other diseases [12]–[18].

A complementary diagnostic indicator of drug response is mRNA expression. This approach is less biased than candidate-gene approaches because it uses global transcriptional signatures of cells in the untreated state to predict drug response. Specifically, previous work, exemplified by Staunton *et al.*, on the NCI-60 panel – a collection of 60 tumor cells lines of different tissue origins that has served as the primary cellular model of cancer genomics [19] – demonstrated that steady-state mRNA expression could be used to predict the sensitivity of cancer cell lines to anti-neoplastic drugs [20]. In related work, others have shown that different physiological correlates besides gene expression, such as protein abundance, may be used to predict drug response [21], [22]. More recently, studies have exploited a collection of genotyped mammalian cell lines to interrogate drug response, and others have performed expression profiling of mammalian cells in response to drug treatment [14], [17], [23]. We sought to perform a drug-response prediction analysis in a well-controlled system. We have previously studied segregating variation in a panel of 104 genotyped segregants derived from a cross between a laboratory strain (BY) and a wild isolate (RM) of *Saccharomyces cerevisiae* [24]–[27]. In the present study we compare how well drug response is predicted by two types of variables—expression and genotype, test the potential improvement in drug-response prediction by combining more than one variable type, and evaluate why one variable type is more predictive than another for a given drug or class of drugs. Our approach affords an opportunity to assess the predictive power of transcripts versus genetic markers in a simple model system, and may serve as an initial point of departure for future analogous studies of personalized medicine in humans.

## Results

### Drug response can be predicted from transcript levels in untreated cells

**Figure 1:**
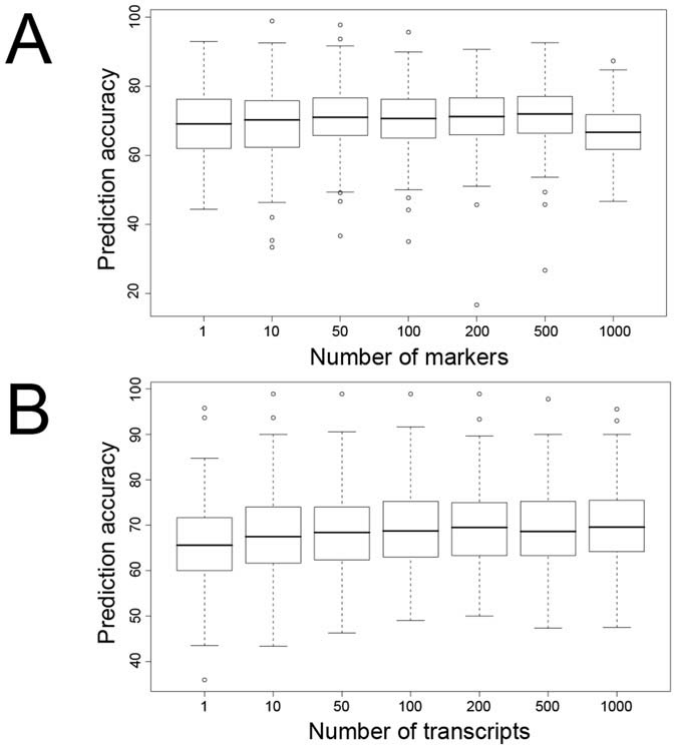
Summary of marker-based and transcript-based prediction algorithms. Box plots representing the distribution of prediction accuracies for all SMPs plotted against number of features selected for prediction. (A) Results of marker-based expression. (B) Results of transcript-based prediction.

We sought to predict the response of each segregant to each small-molecule perturbagen (drug), or SMP, from patterns of gene expression measured in a neutral (i.e., SMP-free) medium. We classified each segregant as sensitive, resistant or partially resistant to a given SMP according to its final yield in that SMP; 225 SMP responses were tested (this represents 89 SMPs, with multiple responses to some SMPs measured at different time points and concentrations). The gene expression levels of the segregants classified as sensitive or resistant were used to train a support vector machine (SVM) [28]. SVMs are very powerful at classifying multidimensional data, and therefore should give us the ability to predict both Mendelian and genetically complex SMP responses. For each SMP, we used a feature selection algorithm within each fold of the cross-validation approach (see Methods) to rank the genes according to their individual contribution to the ability to predict segregant SMP responses. We then trained support vector classifiers using 1, 10, 50, 100, 200, 500, and 1000 highest ranked gene(s). The classifiers were used to predict sensitive/resistant status of segregants not included in the training set using a cross-validation approach (**Methods** **and** **Data S1**). We found that the support vector classifier trained on 1000 genes had the greatest average predictive power, correctly predicting the SMP response of 69.7% of the segregants on average for the SMPs considered, although it should be noted that the differences between 50, 100, 200, 500 and 1000 highest ranked genes are negligible (**Figure 1**). The prediction accuracy for individual SMPs varied from near 100% to near chance. We compared this classifier to a naïve mode classifier, using the same cross validation as with the SVM, which calls all segregants in the test set sensitive or resistant according to the category that occurs more frequently in the training set (this provides a better comparison of the predictive value of expression information than does 50∶50 random classification). Taking the prediction accuracy from the best performing set of features for each compound, the SVM outperformed the mode classifier on average (74% vs. 64%) and equaled or outperformed it for all but one SMP considered (the single instance was well within the standard deviation of the SVM performance). Instances where the mode classifier performed well reflect unequal distributions of sensitive and resistant segregants. Performance was very robust when the algorithm employs classifiers trained on the 50 through 1000 most highly ranked genes. Performance decreased slightly for the classifier with 10 genes, and dropped more appreciably for the classifier trained using the single most highly ranked gene (although performance still remained above chance). This decline in performance is likely due to insufficient or noisy information when too few genes are used.

### Comparison of transcript- and marker-based prediction of drug response

After demonstrating our ability to predict SMP response using steady-state expression alone, we sought to compare these results to prediction based on genotypes. Linkage analysis is dependent on an association between a genotyped marker and a phenotype, in our case sensitivity or resistance to an SMP. Any response to an SMP that significantly links to a marker should therefore be well predicted by that same marker. We first used a much simpler algorithm than the one described above, wherein the genotype at the single most correlated marker was used to predict sensitivity or resistance. We repeated this process in a leave-one-out fashion for all classified segregants. Because we are using the most correlated marker, the response to SMPs exhibiting strong linkage should be easier to predict than response to SMPs exhibiting weak linkage or no linkage. On average we correctly predicted SMP response with 69% accuracy, but, as expected, prediction accuracy was good (75%) when a strong linkage signal was present (lod ≥4) and poor (55%) otherwise. When no strong linkage signal was present, the prediction accuracy was worse than the performance of the mode classifier, highlighting that in these instances the single most correlated marker offered almost no information to perform classification.

**Figure 2:**
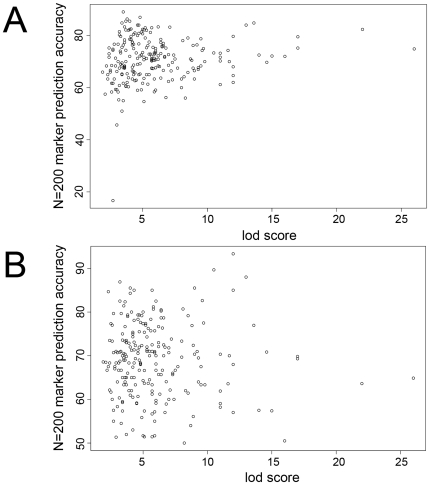
The relationship between linkage and prediction accuracy. Scatter plot of prediction accuracy (in percent) of (A) transcript-based prediction or (B) marker-based prediction versus SMP lod score when the 200 best features are selected.

We further sought to examine our ability to predict more complex SMP responses (those without strong linkage results). We trained support vector classifiers using 1, 10, 50, 100, 200, 500 and 1000 highest ranked marker(s). We found the support vector classifier trained on the 500 highest-ranked markers to have the greatest predictive power overall, correctly predicting the SMP response of 71.7% of the segregants on average for the SMPs considered (**Figure 1**). Performance was very robust in the range of 50–500 highest-ranked markers, but worsened at 1000. A deterioration in classifier performance when more than 500 markers are considered is a result of classifier overfitting. After removing two outliers (alpha factor and niguldipine, which each show linkage with lod >40 and are nearly perfectly predicted using both transcripts and markers), we correlated prediction accuracy with linkage (e.g., lod score) when the prediction algorithm utilizes either the 200 highest-ranked features (**Figure 2**) or the single highest-ranked feature (**Figure S1**). The correlation between marker-based prediction accuracy and linkage is modestly positive (r = 0.13) when using 200 features and, as expected, significantly greater when predicting on the single best feature alone (r = 0.48).

**Figure 3:**
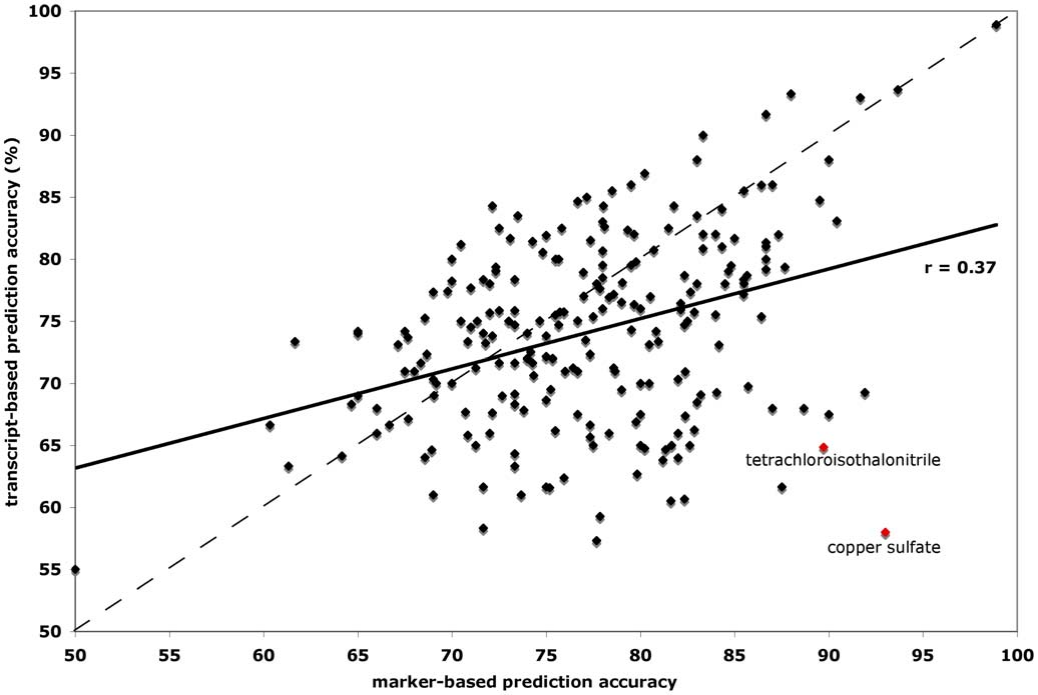
Head-to-head comparison of marker-based prediction and transcript-based prediction. Plotted are maximum predictive accuracies (in percent) of transcript-based prediction (y-axis) versus marker-based prediction (x-axis). Regression line is solid black; the diagonal (x = y) is dashed black; red points denote SMPs described in the main text as that are well predicted by genotype but poorly predicted by expression.

A direct comparison of transcript-based prediction and marker-based prediction is presented in **Figure 3**. For classifiers trained on feature sets ranging from 1 to 1000 features, we plotted the maximum prediction accuracies of transcript- and marker-based prediction for each SMP; more or fewer features are required for maximum accuracy depending on the SMP. The plot reveals that both prediction algorithms perform equally well for a large number of SMPs, but are weakly correlated (r = 0.37) with each other. This weak correlation suggests that there is non-overlapping biological information embodied by transcript levels as compared to genotyped markers. However, some SMP responses (the on-diagonal points in **Figure 3**) are equally well predicted by transcripts and by markers, demonstrating that both are providing equivalent amounts of information. It is possible that the information being provided by the two sets of predictors is redundant, resulting from the fact that expression differences among the segregants arise due to the underlying genetic variation. For example, consider the response to the SMP alpha factor. Alpha factor is a 13 amino acid pheromone secreted by yeast cells of the alpha mating type in the presence of yeast cells of the opposite **a** mating type; **a** cells arrest in the presence of alpha factor because they express the sensitizing alpha-factor receptor *STE2*. Genotype at the mating type locus and expression of *STE2* are completely redundant in this case where sensitivity is determined by the presence or absence of the drug target. A clinical analogy would be clinical efficaciousness of EGF-receptor antagonists (e.g., gefitinib) in the 10% of patients with lung cancers that express sensitizing alleles (somatic deletions and point mutations) of the EGF receptor [29]. Genotyping the EGF receptor stratified patients into drug-responsive and drug-unresponsive cohorts, and EGF receptor expression levels correlate with drug sensitivity.

22 SMPs are better predicted (>15% percent improvement) by markers than transcripts, while no SMPs are better predicted by transcripts than markers by the same margin. In fact, only 6 SMPs are better predicted by transcripts than markers by 10%, and of these none by greater than 12.2% (**Figure 3**; **Figure S1**). One SMP for which genotype is much more predictive is tetrachloroisophthalonitrile, an uncoupler of oxidative phosphorylation. The maximum predictive power of expression for tetrachloroisophthalonitrile is 65% considering the 500 most predictive transcripts, while the maximum predictive power of genotype is 90% considering only the single most strongly linked genetic marker. In previous work we showed that a non-synonymous mutation in the gene *PHO84*, which encodes a high-affinity inorganic phosphate transporter, alters sensitivity to tetrachloroisophthalonitrile [27]. We also showed that the quantitative trait locus (QTL) on chromosome 13 that contains *PHO84* is a linkage hot spot that affects response to 25 SMPs [27]. The above-mentioned SNP in *PHO84* also alters to a lesser extent sensitivity to the chemically similar SMP pentachlorophenol, which suggests that greater genetic complexity underlies the physiological response of cells to pentachlorophenol. The maximum predictive accuracy of expression for pentachlorophenol is 69% considering the 500 most predictive transcripts, while the maximum predictive power of genotype for it is 90% considering the single most linked genetic marker. Another example of more accurate genotype-based prediction is response to copper sulfate (CuS0_4_). The maximum predictive power of expression for copper sulfate is 58% considering the 10 most predictive transcripts, while the maximum predictive power of genotype is 93% considering only the single most linked genetic marker. Linkage analysis has shown that a marker near *CUP1*, which encodes a copper-binding protein that mediates resistance to copper stress, segregates with copper sulfate resistance; *CUP1* is also subject to copy-number variation between strains [27]. The inability of transcripts to predict SMP response may occur when genetic variation does not perturb expression levels under neutral (drug-free) conditions, especially in the case of stress-responsive genes, and therefore does not manifest a steady-state expression signature that would enable transcript-based prediction.

Next we considered cases where transcript-based prediction out-performs marker-based prediction. Expression outperforms genotype for 80 SMP response predictions above the diagonal in Figure 3. This improvement may be due to chance or to expression signatures caused by genetic factors. However, as mentioned above, there are no SMP responses for which expression-based prediction is 15% more accurate than genotype-based prediction (**Figure 3**). This result is consistent with expectation given that expression differences are ultimately explained by genetic differences in these yeast strains. However, there are 6 SMP responses where expression-based prediction outperforms marker-based prediction by 10% or more. In these cases, expression may be a better predictor of SMP response than genotype because several unlinked polymorphisms, possibly in transcriptional regulatory genes, could affect steady-state expression of multiple genes in the pathway modulated by the SMP. Additionally, in cases where transcript-based prediction outperforms marker-based prediction by at least 10%, the average LOD score is 5, while in the reverse case it is 8.5. This is consistent with the idea that transcripts may be valuable when sensitivity or resistance has a complex genetic basis with many minor-effect variants rather than one major-effect variant.

### Using both transcript and marker data improves prediction ability for SMPs

We next asked whether combining both transcripts and markers into a single prediction algorithm would improve our ability to predict SMP response. First, we looked at the best prediction accuracy across all feature sets of both marker- and transcript-based prediction. In 80 out of 226 SMP responses tested, the best transcript-based prediction outperformed the best marker-based prediction, with an average improvement in accuracy of 4.8%. Interestingly, there are no distinguishing mechanistic characteristics of this group of 60 SMP responses, (which, in some cases, includes the same compound tested at multiple concentrations or at multiple time points); in other words, they are structurally diverse and target a wide array of cellular processes. This suggests that transcript information can provide additional predictive information above genotype data alone.

As a second test, we created a combined set of features that included all transcripts and markers, totaling over 9,000 features. We repeated the above-described process of selecting the best 1, 10, 50, 100, 200, 500 and 1000 features, and then used them to train a support vector classifier. The set of 500 features performed best on average with an accuracy of 72%, essentially the same as the marker-based prediction using the same number of features (71.6%). Interestingly, genotyped markers comprised over 95% of all selected features, with many (60) SMP response predictions based solely on marker features. These results are consistent with the observation that genotyped markers provide most of the information used in SMP response prediction. However, when transcripts are selected, they often encode gene products involved in biological processes affected by the SMP. For example, carbonylcyanide p-trifluoromethoxyphenylhydrazone (FCCP) is a proton ionophore that depolymerizes the mitochondrial membrane potential [30]. At least three QTL determine drug response to FCCP in this cross [27]. Expression of two genes, one encoding a component of the vacuolar ATPase (*YDL185W*) and the other a component of the F1-F0 ATP synthase (*YMR064W*), improves FCCP response prediction. The differing information provided by the best combined set of features versus the best set of markers suggests that valuable insight may be gained from using both steady-state transcript levels and genotyped markers. However, combining both transcripts and markers into a single set of features rarely performs better than taking the best of marker-only or transcript-only prediction accuracy, possibly due to the added noise of too large a set of features.

## Discussion

We and others have previously shown that naturally recombinant yeast strains provide a model for the study of therapeutically relevant complex traits (i.e., small-molecule drug response) [26], [27], [31]. Here we have shown that in addition to serving as a model for complex traits a panel of 104 genotyped and expression-profiled yeast strains may also serve as a model for personalized medicine. In the present study, we used a pattern recognition algorithm to accurately predict the sensitivity to small molecules of individual segregants from both steady-state transcript levels in untreated cells and genotyped markers. We observed that markers are slightly more predictive overall, but much better in a few cases. For example, resistance to polychlorinated phenols due to a polymorphism in the high-affinity inorganic phosphate transporter *PHO84* is poorly predicted by transcripts but accurately predicted by markers. It should be noted that we predicted compound response from steady-state mRNA expression levels not only when inheritance of both compound response and expression levels is Mendelian, but also in cases when inheritance of both compound response and expression levels is genetically complex. Moreover, we accurately predicted SMP response in cases when there existed strong linkage between a marker and SMP response (lod >4) as well as when no strong linkage was present (lod < 4).

Transcript-based prediction performs similarly to marker-based prediction for most SMP responses, and thus expression profiles provide a useful proxy when genotypes are not available. Expression may sometimes be a better predictor of compound response than genotype because expression can integrate many genetic changes, and may therefore reflect the overall physiological state of the cell rather than just the effect of one locus. On the other hand, expression may be a poorer predictor of compound response than genotype in cases when transcript levels of untreated cells is uncorrelated to transcript levels of drug-treated cells. Gene expression may capture the same information as genotypes for several reasons. First, a polymorphism may affect both gene expression and compound response independently (pleiotropy), with the expression levels providing a read-out of the inheritance at the locus. Second, a polymorphism that affects compound response may be linked to a different polymorphism that affects gene expression. The third and most interesting case involves polymorphisms that affect the expression of drug targets or other genes that function in the pathways that are involved in SMP response; in this case the expression changes provide direct functional information. Further functional studies are needed to distinguish these possibilities and quantify the prevalence of each.

We observed that expression provided little predictive power over genotype alone. In our system, genotype largely determines both expression levels and drug response; environmental conditions were kept constant during expression experiments, and only differed on the basis of SMP treatment in drug response experiments. We expect that gene expression will provide considerable additional predictive power when environmental variation is present, for example in human patients who will differ in diet, drugs taken, and other factors. This study demonstrates the benefit of having multiple sources of data in understanding complex pharmacogenomic traits.

## Methods

### Chemoprediction algorithm

Segregants were classified as sensitive or resistant based on the standard deviation from zero of each segregant's six replicate growth values. If a segregant's average growth rate in the presence of a given SMP was at least one standard deviation below zero it was considered sensitive to that SMP; if the average growth rate was a standard deviation or more above zero it was considered resistant. Segregants with standard-deviation ranges overlapping zero were not classified, and were removed from the analysis. Segregant growth rates at various time points were available for some of the 92 SMPs surveyed, providing a total of 333 sets of segregant growth rates. To be able to determine a pattern between gene expression and sensitivity or resistance to a given SMP, a sufficient number of “sensitive” and “resistant” segregants are needed. Therefore only growth rate sets with at least 10 sensitive and 10 resistant segregants were treated, eliminating 107 growth rate sets.

Before applying the prediction algorithm, we reduced noise in the data by ranking each of the segregants' genes' association with drug sensitivity. For each of the 226 sets considered, a stratified 10-fold cross-validation scheme was used to select features and train support vector classifiers, and to test the classifiers. This involves random division of the data into ten similarly sized parts, each with a classification profile (in this case, the ratio of sensitive to resistant segregants) approximately representative of the full data set; one part is kept aside for testing the classifier, and the remaining nine subsets are used for feature selection and then training; the full selection/training/testing process is carried out ten times using a different portion of data for testing each time. Feature selection – in our case selecting the most relevant genes for segregant response to an SMP – was performed to reduce noise in the data and hopefully make any pattern more readily identifiable. The feature selection algorithm, performed within each fold of the cross-validation scheme, used a support vector machine (SVM) [28], a pattern-recognition or machine-learning algorithm, to weight each gene according to the strength of its relationship with the segregants' sensitivity/resistance to the SMP. (Note that this SVM is independent of the support vector classifier described below, which we use in the second stage of prediction.) The features were then ranked according to the square of the weight assigned by the SVM, with the greatest square ranked highest, and only a specified number of the top-most genes were used for training. Sequential minimal optimization (SMO) [32]–[34] with an RBF kernel, a machine-learning algorithm, was used to train a support vector classifier. For each run through the 10-fold cross-validation, the SMO parameters were optimized and then applied to the segregants' chemosensitivity classification and the selected gene expression data. The classifier was then used to predict the chemosensitivity of each segregant in the test portion, and the results were compared with the actual chemosensitivity to determine classifier performance for that run. The entire process is repeated until each of the 10 subsets has been used for testing. The accuracy quoted is the percent of correctly classed instances in the test portion averaged over all ten runs. Support vector classifiers were trained using the 1, 10, 50, 100, 200, 500 and 1000 highest ranked gene(s). Computation for the training, testing and analysis was carried out using algorithms from Weka (Waikato Environment for Knowledge Analysis) [35] and the Java programming language.

## Supporting Information

Figure S1 (0.73 MB TIF)

Data S1 (0.24 MB XLS)
