# Supplementary figures and images for: Using Expression and Genotype to Predict Drug Response in Yeast
Source: PLoS One. 2009 Sep 4;4(9):e6907. doi: 10.1371/journal.pone.0006907 (PMC2731853; doi:10.1371/journal.pone.0006907)

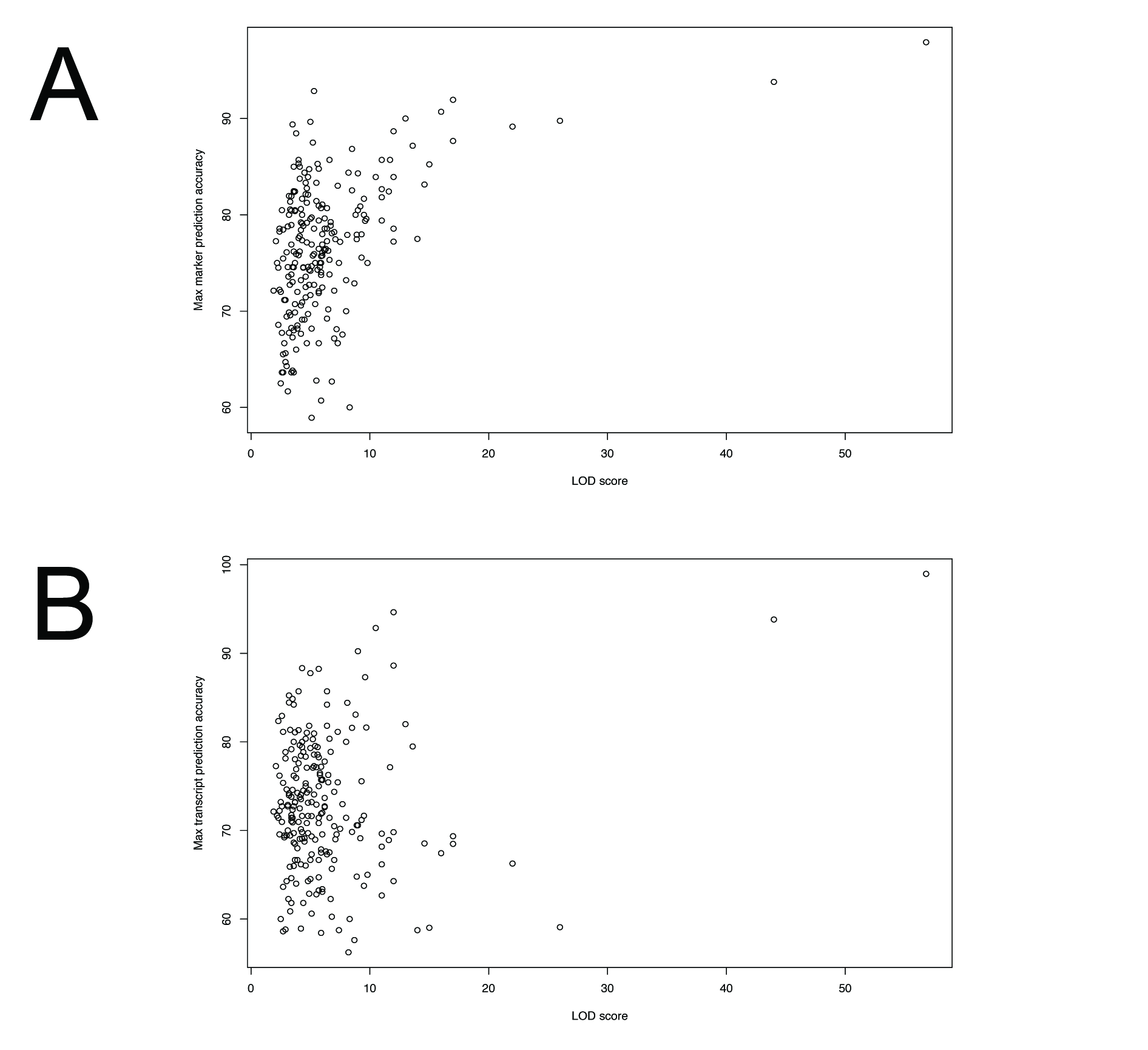

Supplement: Figure S1 — (0.73 MB TIF) [file pone.0006907.s001.tif]
